# Qualitative insights from an online community-based exercise intervention for persons living with HIV

**DOI:** 10.3389/fresc.2025.1602007

**Published:** 2025-08-25

**Authors:** Francisco Ibáñez-Carrasco, Kiera McDuff, George Da Silva, Ahmed M. Bayoumi, Soo Chan Carusone, Mona Loutfy, Ada Tang, Puja Ahluwalia, Lisa Avery, Kelly K. O’Brien

**Affiliations:** ^1^ Dalla Lana School of Public Health, University of Toronto, Toronto, ON, Canada; ^2^ Department of Physical Therapy, Temerty Faculty of Medicine, University of Toronto, Toronto, ON, Canada; ^3^ Realize, Toronto, ON, Canada; ^4^ MAP Centre for Urban Health Solutions at the Keenan Research Centre in the Li Ka Shing Knowledge Institute, St. Michael’s Hospital, Toronto, ON, Canada; ^5^ Department of Medicine, University of Toronto, Toronto, ON, Canada; ^6^ Institute of Health Policy, Management and Evaluation (IHPME), Dalla Lana School of Public Health, University of Toronto, Toronto, ON, Canada; ^7^ McMaster Collaborative for Health and Aging, McMaster University, Hamilton, ON, Canada; ^8^ Women’s College Research Institute, Toronto, ON, Canada; ^9^ School of Rehabilitation Sciences, McMaster University, Hamilton, ON, Canada; ^10^ Biostatistics Department, Princess Margaret Cancer Centre, University Health Network, Toronto, ON, Canada; ^11^ Rehabilitation Sciences Institute (RSI), University of Toronto, Toronto, ON, Canada

**Keywords:** HIV/AIDS, exercise, interviews, community-based research, qualitative research

## Abstract

**Introduction:**

Online community-based exercise (CBE) is a rehabilitation strategy that can promote health outcomes among people living with HIV. We aimed to describe experiences implementing a community-based exercise (CBE) intervention with adults living with HIV.

**Methods:**

We conducted a longitudinal qualitative descriptive study involving interviews with adults living with HIV and persons implementing an online tele-coaching CBE intervention. Leveraging community-based research principles, the intervention aimed to improve physical activity engagement and health outcomes through online individualized coaching, online YMCA resources, and wearable fitness technology. We analyzed interviews with adults living with HIV and representatives involved in CBE implementation at baseline (month 0), end of intervention (6 months), and end of follow-up phase (12 months).

**Results:**

Thirteen adults living with HIV and eight representatives involved in CBE implementation participated in the study (43 interviews total). Key themes included the “four Cs”: Cost, Care, Comfort, and Convenience that encapsulated participants’ perceptions of financial barriers, need for holistic healthcare integration, importance of stigma-free and emotionally supportive environments, and accessibility of health interventions.

**Discussion:**

Results underscore the critical role of inclusive and adaptable exercise programs in addressing the complex needs of individuals with chronic, episodic conditions such as HIV, and the value of participatory, community-driven methodologies in designing effective and equitable health interventions.

**Clinical Trial Registration:**

NCT05006391

## Introduction

In this manuscript, we examine the strengths and weaknesses of a community-based research methodology used to implement an online community-based exercise (CBE) intervention (the intervention) with adults living with HIV. We discuss the rarely reported impacts of CBE implementation on participants and researchers and implications for future community-based research.

An online community-based exercise (CBE) intervention is a digital health strategy designed to promote physical activity among specific populations, such as individuals living with HIV. It involves structured exercise programs delivered through online platforms, enabling participants to engage in physical activity within their communities while accessing virtual support and resources. These interventions often include personalized exercise sessions with certified trainers, access to online group classes, provision of exercise equipment, and the use of wearable physical activity monitors to track progress. The goal is to enhance health outcomes by making exercise more accessible and tailored to the needs of the community (1–3).

In HIV care, aging individuals face unique health challenges, making physical activity crucial for their well-being. Regular physical activity can enhance quality of life and independence, offering a distraction from their condition (4–6). However, stigma, lack of customized programs, and the need for accessible, affordable interventions hinder engagement in physical activity (7, 8). Advances in antiretroviral therapy (ART) have transformed HIV into a manageable condition, yet individuals face physical, mental, and neurological disabilities (9) and economic and workplace participation challenges (10). These chronic conditions strain healthcare systems, highlighting the need for comprehensive disability management within HIV care (11).

Community-based exercise (CBE) programs conducted outside conventional research settings align with community-based research goals, making interventions more accessible, especially for individuals with disabilities such as HIV (12). CBE programs focus on fostering health, minimizing costs, and seeking community support, addressing the complex needs of aging HIV populations (3, 13). However, in-person CBE programs face logistical issues, stigma, and costs (2, 14). To address these challenges, we designed an online CBE intervention leveraging technology, individualized coaching, and access to a YMCA online platform. Our goal was to assess the implementation of this intervention for its ability to reduce disability and enhance health outcomes among adults living with HIV (15). We aimed to do this in a community-based manner. In an earlier phase of work, we identified the complex interaction between environmental, personal, and organizational factors influencing online CBE implementation with adults living with HIV (16). In this study, we conducted a reflexive thematic and contextual analysis, exploring experiences engaging in the online CBE intervention and implementation process from the perspectives of participants and implementing members, focusing on the impact of our community-based research strategy.

## Materials and methods

### Study design

We conducted a prospective longitudinal intervention study using implementation science approaches involving a 6-month intervention phase followed by a 6-month independent exercise follow-up phase. The study was based on community-based research principles, through a long-standing partnership with the YMCA, embedding technology throughout the intervention during the COVID-19 pandemic. Our research team included over 10 members, each contributing distinct expertise: principal investigators provided strategic oversight; research coordinators managed day-to-day operations; co-investigators contributed domain-specific insights (e.g., physical activity, HIV care, and qualitative methodologies); postdoctoral fellows assisted with data analysis and manuscript preparation; and community ambassadors ensured alignment with participant needs and community values. This collaboration enriched the study design, data collection, and analysis, ensuring a rigorous approach to understanding the intervention's impact within this population.

### Ethics

This study was approved by the University of Toronto Health Sciences Research Ethics Board (Protocol no. 40410). All participants provided informed, signed, or verbal consent to participate in the study, documented on a consent form.

### Intervention

The online CBE intervention involved two phases. In the initial 6-month phase, participants were asked to exercise thrice weekly, including aerobic, strength, balance, and flexibility training, supervised biweekly by a personal trainer (synchronous online session), and attend monthly online group educational sessions. In the second 6-month phase, participants were asked to continue with thrice weekly exercise independently. Throughout both phases, participants were provided with an online YMCA membership and wireless physical activity monitor (Fitbit) to track their physical activity. Participants engaged in weekly Fitbit syncing and completed bimonthly and weekly online questionnaires, and a personal trainer from the YMCA conducted bimonthly online fitness assessments. The study involved significant preparation, recruitment, and equipment delivery during COVID restrictions. The 6-month intervention phase occurred in a staggered manner between 23 October 2021 and 9 June 2022 followed by a 6-month independent phase of exercise.

Our study also employed a community-based research lens to analyze the intervention's deployment, with further analysis guided by the RE-AIM framework (17). We recruited adults with HIV and representatives involved in CBE implementation for qualitative interviews to identify strengths and challenges in implementing the CBE intervention.

### Community-engaged approach

The study team included research coordinators, principal investigators, co-investigators, coaches, a postdoctoral fellow, and ambassadors from diverse communities. Details of the study protocol have been previously published (15). In this paper, we specifically focus on exploring the implementation process using qualitative data collected through interviews with participants living with HIV and representatives involved in implementation.

### Participants and recruitment

We included adults who self-identified as living with HIV and considered themselves medically stable to engage in a 12-month exercise intervention study. We also included representatives involved in the CBE implementation, including (but not limited to) personal trainers, researchers, and clinicians involved in HIV or CBE.

We purposively recruited a subsample of participants living with HIV from the larger online CBE study and representatives involved in CBE implementation (e.g., personal trainers) to participate in this qualitative study. Invitations were extended via email by a postdoctoral fellow and a woman with expertise in qualitative research who conducted study intake and baseline assessments. The research team had no prior relationship with the participants prior to study commencement. We specifically recruited women living with HIV to achieve gender diversity in the sample. Participant selection prioritized gender diversity, reflecting our prior research highlighting additional barriers faced by women and gender-diverse individuals in exercise interventions.

### Data collection—interviews

We conducted a series of one-on-one online (Zoom) interviews at baseline (0 months), post-intervention (6 months), and post-follow-up (12 months) with participants living with HIV. In the interviews, we examined participants’ experiences, levels of engagement, and the perceived impact of the intervention.

Specifically, we focused on experiences with the online CBE intervention, the impact of tele-coaching, goal setting, mindset, adherence, social support, and stigma. Using a semi-structured interview guide, we asked about (a) experiences with exercise, (b) anticipated benefits of exercise (initiation), (c) perceived impact of CBE [including prescribed exercise, peer-support, use of a specific exercise tracking device (Fitbit), and optional self-management sessions on physical activity and health over time (post)], and (d) maintenance in exercise (end of study). We also explored how the intervention's design, activities, and quantitative fitness assessment findings aligned with participants’ lived experiences. We paid attention to the use of technology on the impact and engagement in exercise. In addition, we explored the influence of social support, stigma, and personal attributes on the impact and level of engagement in exercise. All interviews were audio recorded and transcribed verbatim. (Supplementary File S1—interview guide). We administered a demographic questionnaire to describe personal, health, and HIV characteristics of participants living with HIV. Participants were provided with a $30 electronic gift card as a token of appreciation for participating in each interview.

### Analysis

We conducted a “reflexive thematic analysis” and a “contextual analysis” to focus on experiences of CBE implementation. Reflexive thematic analysis (18–20), guided by phenomenology and grounded theory, emphasizes the researcher's active engagement with the data and is widely used in health-related research (21). Contextual analysis aligns research with local needs and values, fostering skill enhancement, health improvements, policy influence, and knowledge generation (22, 23). This method is utilized in research on physical activity (24–27).

For our intervention, context encompasses relationships among participants, research staff, ambassadors, and coaches, as well as the online environment. Social and online spaces intricately define interactions, influenced by physical and online contexts (28–30). We conducted a thematic analysis to identify themes such as stigma, healthcare access, and social support among people living with HIV. We used an inductive approach to code line-by-line interview text, highlighting experiences with the online CBE intervention. Lead author (FI-C) read and coded all the transcripts, and co-authors (KO’B, KM, SC) read and coded a subsample of the transcripts. The analytical team met to discuss overall impressions of the interview transcripts as they related to experiences with the online CBE intervention and the key themes that emerged from the analysis. By integrating contextual analysis, we examined how these themes were influenced by historical policies, cultural attitudes, and socioeconomic conditions, providing a richer understanding of participants’ experiences.

NVivo 14 was used to organize coded excerpts of the interview transcripts at the initial, mid, and end stages. Results were considered in relation to research meeting notes and feedback from diverse presentations to academics, clinicians, and community leaders. Specifically, we used meeting notes and feedback from presentations to iteratively supplement and inform our analysis. The contextual analysis included examining the broader context, such as COVID-19 disruptions, funding structure, and implementation activities, to understand how community-based principles were emphasized.

### Sample size

We aimed to recruit 10 participants living with HIV and five CBE representatives to the study. Our prior work indicated this would enable us to achieve our objectives related to the strengths and challenges of implementing CBE (31, 32).

## Results

Thirteen adults living with HIV involved in the CBE study participated in this qualitative study, of which seven participants completed all 3 interviews, three completed 2 interviews, and three completed one interview (30 interviews total) (Table 1). Eight representatives involved in CBE implementation participated in the study, of which two participated in all 3 interviews, one completed 2 interviews, and five completed 1 interview (13 interviews total) (Table 2). These included fitness personnel (coaches), researchers, and clinicians. The interviews were conducted between 8 October 2021 and 6 April 2023. Interviews were approximately 60–90 min in duration.

**Table 1:** Table of participation—adults living with HIV (*n* = 13).^a^

Adults living with HIV (*n* = 13)				
Participant #	Initiation of CBE intervention (month 0)	End of CBE intervention (month 6)	End of follow-up phase (month 12)	Total number of interviews
P2	**√**	**√**	**√**	3
P3	**√**	**√**	**√**	3
P4	**√**	**√**	**√**	3
P8		**√**	**√**	2
P9	**√**	**√**	**√**	3
P13	**√**			1
P14	**√**	**√**	**√**	3
P16	**√**	**√**		2
P20	**√**		**√**	2
P22	**√**	**√**	**√**	3
P23			**√**	1
P25	**√**	**√**	**√**	3
P34			**√**	1
**Total number of interviews**	**10**	**9**	**11**	**30**

Bold indicates total number of interviews.

^a^ Participant numbers are not sequential as this represents a subsample from the larger CBE intervention study.

**Table 2:** Table of participation—representatives involved in CBE implementation (*n* = 8).

Representatives involved in CBE implementation (*n* = 8)				
Participant #	Initiation of CBE intervention (month 0)	End of CBE intervention (month 6)	End of follow-up phase (month 12)	Total number of interviews
CBE1	**√**	**√**	**√**	3
CBE2	**√**	**√**	**√**	3
CBE3	**√**			1
CBE4	**√**			1
CBE5	**√**		**√**	2
CBE6		**√**		1
CBE7			**√**	1
CBE8			**√**	1
**Total number of interviews**	**5**	**3**	**5**	**13**

Bold indicates total number of interviews.

### Characteristics of participants

Among the 13 participants living with HIV who took part in an interview, the median age was 58 years (25th, 75th percentile: 45, 63), with 69% over 50 years. Gender distribution was 54% men and 46% women. Participants living with HIV self-identified as Black or African Canadian (54%), South Asian (23%), White (15%), Latin American Hispanic or Latino (8%), and Southeast Asian (8%). The median number of comorbidities was 2, and the median years since HIV diagnosis was 19 (15, 32). Most (11; 85%) had an undetectable viral load. Six (46%) were working full time or part time; one (8%) was a student; three (23%) were retired; one (8%) was on disability; and two (15%) were unemployed/not working but seeking work. General health status among participants living with HIV was rated very good (9; 69%) or good (2; 15%) by most participants. Further details on the characteristics of participants engaged in the online CBE study are previously published (16).

### Interviews results

To summarize the emergent themes, we used four Cs as a prism metaphor to capture how participants viewed their relationship to physical activity in their everyday lives and through this research and online intervention (33). The four Cs metaphor emerged during public online and in-person presentations. We saw the positive audience response to the metaphor, especially from community leaders living with HIV.

**Figure 1:**
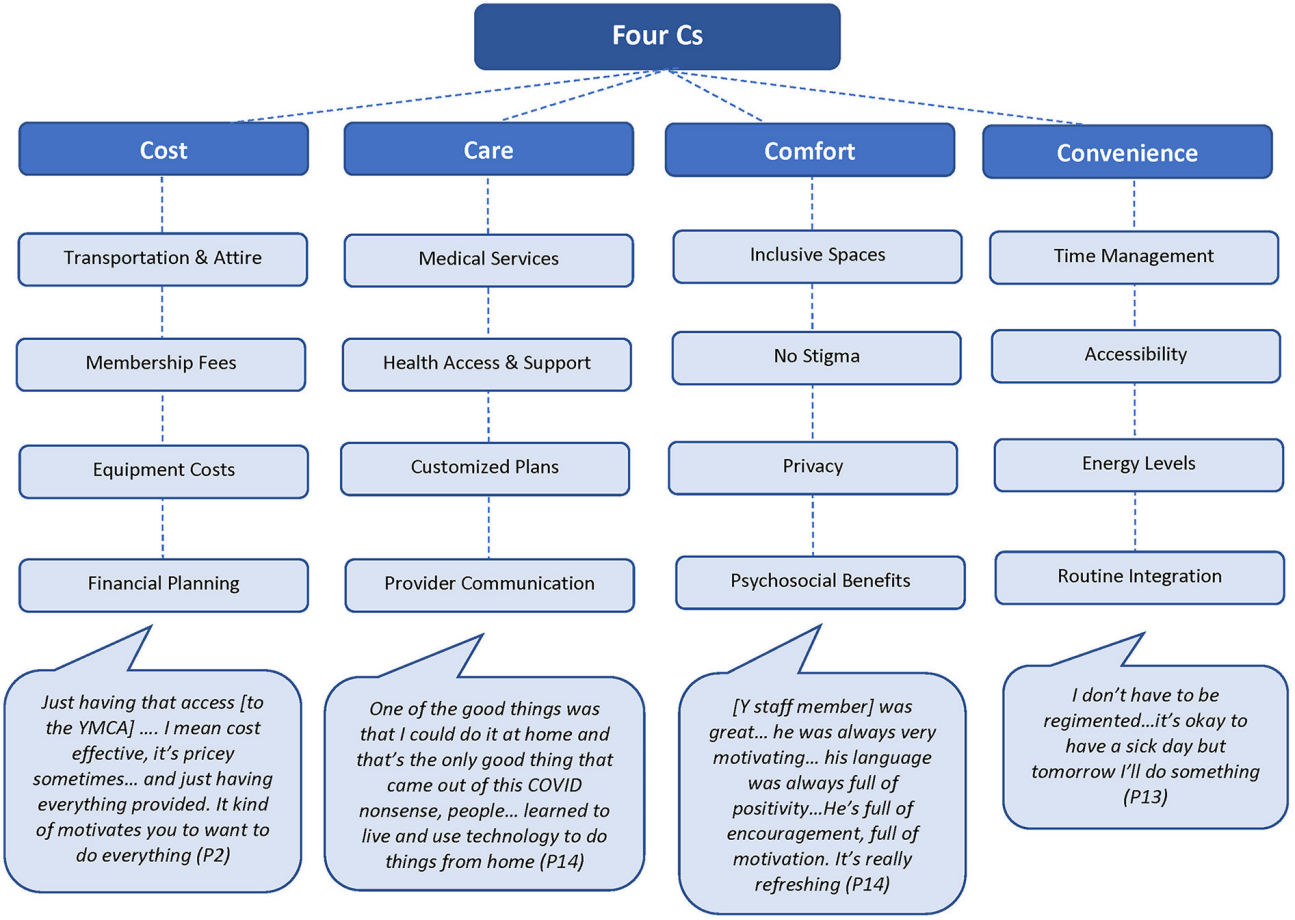
The four Cs: cost, care, comfort, and convenience.

The four Cs metaphor stands for Cost, Care, Comfort, and Convenience. Figure 1 illustrates the four Cs of an intervention—Cost, Care, Comfort, and Convenience—each with key subsidiary ideas: financial aspects, medical services, inclusive environments, and accessibility considerations, respectively. Cost concerns include financial burdens and societal pressures. Care encompasses the need for comprehensive health services, the need for tailored physical activity plans, and the imperfect communication about physical activity between health care providers and patients. Comfort pertains to psychosocial benefits, advocating for non-stigmatized, inclusive physical activity services, social and mental health services, as well as a sense of belonging and intact privacy. Convenience addresses the ease of accessing any kind of supporting, social, and/or mental health services available to a person, influenced by privilege and gender roles. Throughout this section, we interpret the themes as they relate to the broader literature. In these results, we focus on perspectives of adults living with HIV; while interviews with representatives involved in CBE implementation informed our interpretation. For the supporting quotes, we indicate the participant number, the participant group (participant living with HIV; representative involved in CBE implementation), and time of intervention (initiation of CBE; end of CBE intervention; end of follow-up phase).

### Cost

Considerations about the cost of exercising in gyms, equipment, clothing, and commonly believed beneficial supplements and vitamins are not new to people living with HIV and other chronic and episodic conditions. Research indicates that economic barriers impact the ability of individuals living with HIV to engage in regular physical activity, despite the well-documented health benefits. In our analysis, we were not surprised to find these complaints and opinions pervasive. People living with HIV may have access to city public gyms and pools, but attending them still requires transportation, and what was deemed appropriate attire is an additional financial burden (34, 35). The fact that we partnered with the YMCA in downtown Toronto may have also had an effect on the participants’ consideration of cost. In that regard, the access to a paid gym facility in the CBE intervention is “nice to have” but may not necessarily inspire certainty for a long-term costly commitment.

I was a paying member there [YMCA] years ago, and I can remember when the old YMCA was torn down, and the new one was put up… I looked at the facility and how luxurious… I don’t know what they called it, ‘corporate membership’ where you get to use the area with the whirlpool and that… And I just thought that really runs counter to what I think the Y’s mandate should be. This is giving like exclusive privileges to someone who’s got the money to afford it. It kind of rubbed me the wrong way because there were a lot of thresholds that one had to meet to get a reduced membership at the Y. I find affordability is an issue that I think the Y should re-look at. Now of course they need to find funding from somewhere. They’re not there to do this for free. It costs them… So perhaps sourcing funding from somewhere that has a mission you know to cater to this community. Maybe that would be something to pursue. (P9; participant living with HIV, end of CBE intervention)

The quote reflects a well-established need in the field of HIV management to address and reduce the poverty experienced by many people living with HIV. As Kalichman emphasized in “Ending HIV Hinges on Reducing Poverty” (36), alleviating poverty is crucial for effective HIV management and improving the overall well-being of those affected. In this passage, the metaphors of cost and convenience merge, the participant exposed the inherent contradiction of doing exercise while all the perks of research participation are provided, and the unlikelihood that they will keep it up after the CBE intervention ends.

Q: If there was one aspect of the study that worked best for you, what would that be?

R: […] So just having that access … It’s not something that I would have thought of—going to sign up for the YMCA. I mean cost effective obviously, it’s pricey sometimes… And just having everything provided. It kind of motivates you to want to do everything. I still have all the stuff that I picked up, and I utilize them, not as often as I should, but for the most part, I do use everything that was provided to me to be able to participate in the activities that I’m doing. So that was very I guess beneficial. Even now if we don’t go to the YMCA, I still have access to the [online] platforms. Yeah, just that opportunity to participate and engage in the activities. (P2; participant living with HIV; end of CBE intervention)

### Care

“Care” is the metaphor we use for people receiving medical and social provision of services. It involves a process of candidacy (37, 38) in seeking and accessing healthcare professionals, treatments, and supportive therapies alongside emotional support, community resources, and informal networks. This holistic approach ensures coordinated, personalized care plans that address both medical and social needs, promoting overall health and well-being. In the Canadian context, basic medical services are covered by federal and provincial governments. Complementary care is out of pocket (39). However, the participants warned us that this cannot be generalized to all persons living with HIV, neither in public health nor in our CBE intervention.

For me, one of the reasons why I don’t join the gym and why I have an exercise bike and all of that is because of finances. Joining a gym means paying and they’re expensive. They’re not cheap. And convenience… if I’m on my way home from work, if the gym or the space to exercise is along the way home. Yes, I can stop and continue. But if I have to get out of my way, then yeah, that’s not going to happen. One of the good things was that I could do it at home and that’s the only good thing that came out of this COVID nonsense, people… learned to live and use technology to do things from home kind of thing. That was the good thing. Bringing it out of research and into the real-world kind of thing, I mean working with ASOs [AIDS service organizations] so that clients who are accessing this service through ASOs don’t have to worry about paying for it and still get the support of a trainer kind of thing. But that’s all depending on the agency, what they can provide kind of thing. But it would be a good thing to have this benefit. (P14; participant living with HIV; end of follow-up phase)

The participant obliquely critiques the choices made by the research team and the pervasive and prevailing narrative of doom and gloom in the HIV movement. In Canada, patients may not frequently discuss exercise with their physicians for various reasons, including prioritizing immediate health concerns, perceiving the physician's role as more focused on medical treatment than lifestyle advice, and limited consultation time, which can lead to prioritizing more urgent health issues (40). Patients might also assume that their physicians are not well-informed about exercise, or they may not feel comfortable bringing up the topic due to communication barriers. Additionally, patients’ beliefs about the benefits of exercise and its relevance to their health condition play a role; if they do not see a direct connection to managing their health condition or comorbidities, they are less likely to mention it (41–45).

### Comfort

“Comfort” is a key psychosocial need for people living with HIV, requiring spaces that do not remind them of their condition or emphasize HIV in their lives, concrete and social spaces, practices, and tools that enhance adherence and linkage with services (46, 47). Many people living with HIV, especially those aging with HIV, feel discomfort due to internalized and anticipated HIV stigma, making both physical and virtual spaces potentially uncomfortable for those with episodic disabilities and comorbidities as a result of anticipated or internalized HIV-related stigma (48) and garden-variety misogyny, ableism, and ageism.

In the following excerpt, the participant living with HIV finds comfort in exercising at home with the online intervention, away from the youth-oriented, appearance-focused environment of public gyms. This home setting eases feelings of ageism and stigma, providing a supportive and inclusive atmosphere. The gentle encouragement they receive at home enhances their psychological comfort and engagement in exercise.

Q: The six Self-Care sessions were at a tricky time for some participants because we had it between 5 pm and 7 pm during the week. Did you get a chance to see any of them or attend?

R: No [I did not have the chance to attend the six self-care sessions]. The timings were always during working hours. It’s not possible. And the sad reality is, many of these programs that are for people who are HIV+, the assumption is people who are HIV+ are on ODSP, they’re bums, they’re doing nothing, they’re sitting at home, they’re idle, they’re wasting their time, and they’re useless. [anticipated stigma] And that’s why everything is set during office hours during the week… That’s an unconscious mindset and the reality is far from the truth… I know so many professionals who are HIV+ and yet they work. They have careers, they have families, they have both. They live exactly normal lives like everybody else. (P14; participant living with HIV; end of CBE intervention)

The start of the conversation about optional free six self-care sessions also cues in the convenience metaphor we explore later in this text.

In many of the interviews illustrated by the excerpt chosen here, the focus shifts from discussing the numerous fitness assessments associated with the evaluation of the intervention to the coach's role as a “healing witness,” which refers to the coach's ability to empathetically observe and support the participant's life experiences without extensive preparation (49–51).

Q: Finally, what did you think of the fitness assessments, your experience doing the fitness assessments with [a Y staff member]?

R: [Y staff member] was great… He was always very motivating… His language was always full of positivity. Even… when I do horrible push-ups… He was motivating. I like him. He’s full of encouragement, full of motivation. He was always very positive. It’s so positive. It’s really refreshing. It’s very nice. (P14; participant living with HIV; end of CBE intervention)

Coaches frequently observed that participants preferred chatting over exercising, likely due to the COVID-19 lockdown and the coaches’ role as emotional support providers. Participants often prioritized emotional comfort over physical exercise, highlighting the need for trauma-informed coaching. Although some trauma-informed practices were implemented, more emphasis might have been necessary. Literature primarily addresses this need in youth, but our intervention suggests its importance for individuals with highly stigmatized conditions such as HIV.

In our intervention, the concept of “comfort” proved to be flexible and included the discomfort and inconvenience associated with the technology used. The wireless physical activity monitor (Fitbit), with its steep learning curve, the need to replace it with a trusted wearable watch, and the requirement to download and understand usage reports, became a focus for both participants and researchers. This highlighted the importance of addressing technological challenges to ensure participants’ comfort and engagement with the intervention.

Q: Is there one part of the study that worked best for you?

R: … Definitely Fitbit… not only having the Fitbit. It helped me to track all my things; the steps, those all sleeping patterns and even weekly… for that report. Those are all in the Fitbit apps. So those things, seeing those all tracked, it gives me… *It satisfied me*. It motivates me. (P16; participant living with HIV; end of CBE intervention)

The one thing I hated the most was this useless Fitbit. Every month there was a problem with it. Every single month there was something wrong. It had a mind of its own. It would go back to being dim. It would go back to something. Like every month I had to go to the app, I had to go to the Fitbit, reset the settings like this is nonsense. The only thing it was good for was to see how many steps I’m doing per day but that’s about it. Other than that, I really don’t use it. I think it’s a waste of my time, and it doesn’t even work properly. (P14; participant living with HIV; end of follow-up phase)

These two interview citations illustrate that convenience and comfort in using the Fitbit varied widely: some participants found it motivating and easy to use, while others faced technical issues and inconsistent support, leading to frustration. These experiences underscore how convenience is influenced by access to reliable technology, effective support systems, and individual circumstances, reflecting broader themes of privilege and gender roles. These complications have been reported in previous studies (52, 53). Technologies and “being seen”: on the one hand, participants in the intervention want to be witnessed, and on the other, they also have apprehensions about being seen through the magnifying and revealing glass of the webcam; it is an ongoing contradiction. Comfort, the provision of spaces without direct judgment, stigmatization, or fewer opportunities to stir the anticipated stigma of persons living with HIV, seems a simple goal in an online exercise Intervention. However, the convenience and engagement of participants in our intervention were subtly but deeply influenced using technologies. In this case, the pervasive and yet invisible aspect of “seeing and being seen online,” which alters mediated human activity. This challenge impacts sustaining engagement and body image. During COVID-19, individuals experienced barriers to virtual classes, including technology competency, space, equipment limitations, lack of personalized guidance, safety management, reduced accountability, social support, privacy concerns, and loss of routine (54).

These results highlight the burden of “being seen” in one's home, with its pressures and limitations, compared to a gym's social dynamics of ageism and ableism. While exercising at home seems private, exercising in front of a webcam carries a loss of privacy (55). The impact of “being seen” is well-documented in telemedicine, where its effect on relationships remains debated (2, 56–59). Overall, knowledge mobilization risks overburdening specific patient groups, leading to participant fatigue and reduced autonomy, making them feel such as captive audiences (60, 61). The quote below reflects the contradiction faced by the participants when using their webcam.

Q: You said that you had to work some things out a little bit when you started with your coach. What kinds of things did you need to work out with the coach?

R: I think it was… privacy that you can maintain some privacy when doing this because you know. I could be pretty uncoordinated… I do think you kind of have to sit down and say is this physically going to work, what type of exercise am I doing, what type of space do I need front, back, sides, up above, how private is it, am I going to be safe in this space meaning can I get to the stuff I need. (P22; participant living with HIV; initiation of CBE)

### Convenience

In our analysis, “convenience” included having the time, opportunity, strength, and energy, as well as a sense of belonging and the easing of stigma, such as body hyperawareness and comparisons in a gym setting. This is particularly important for persons aging with HIV, who often experience body image dysfunctions (62). Convenience also determines whether research activities are genuinely beneficial for participants. Convenience likely outweighs perceived benefits when it comes to physical activity (41, 63). The metaphor of convenience is critical for successful health interventions, as engagement often hinges on how convenient the interventions are.

Convenience is often named as a social determinant of physical activity (64); on the other hand, it also encodes the convenience most consumers/patients might reasonably expect today from a research intervention using a community-based research strategy (65). Making health research interventions convenient is critical for enhancing participation and ensuring sustained engagement, which ultimately leads to better research outcomes (17, 66, 67).

First, let's examine the quotidian use of “convenience.” This first passage states that despite the barriers of access and cost, convenience is a key factor in encouraging individuals to engage in physical activity, especially for women. It suggests that when exercise routines, times, and conditions are convenient, participants are more likely to incorporate them into their daily habits.

Q: It sounds to me that forming the habit is one of the important goals for you.

R: It’s so easy for me to come home from work, and I’m exhausted… There’s going to be days maybe where your shift changes and you’ll be a little bit more exhausted. But that’s a day you just choose to switch your workout… You switch it to work your schedule, and that is taking me a lot of time to wrap my head around… I don’t have to be regimented, and I think everybody that I know that does this kind of workout and things tend to be regimented. I’m like no, it’s okay to have a sick day but tomorrow I’ll do something… It’s okay to not be able to do today if you work late today, do a class tomorrow… getting the mindset will be the most rewarding part because keeping to a routine. I’m also a person that needs to be flexible in that routine because there are some days where maybe I work early. (P3; participant living with HIV; initiation of CBE)

When we consider the broader experience of participants in the intervention, it becomes clear that we may not have fully assessed whether the format was convenient for them. While we believed the CBE intervention was beneficial due to the significant attention and perks provided at no cost, feedback suggests otherwise. An interview excerpt highlights a participant's struggle with the repetitive nature of weekly surveys, leading to survey fatigue. The participant recommended making the questionnaires more engaging and less monotonous to improve the convenience and manageability of the evaluation process. This feedback underscores the need to simplify and vary tasks to reduce mental burden and maintain participant engagement.

A: It’s a lot more work than just getting some good exercise and nutrition and advice… There’s a lot of mental aspect [mindset] that goes into it… I don’t even read them [study questionnaires anymore because I know the questions. I don’t even have to look at what I’m reading. I just put it in because there’s no change. It’s the same. I get the why we must do it… I know it’s because it’s a scientific thing. But it’s so dry… It’s just survey fatigue. (P3; participant living with HIV; end of CBE intervention)

Our objective to achieve multiple goals within a limited time frame, particularly during the COVID-19 lockdowns, created significant pressure. From the researchers’ perspective, we implemented a structured and phased process: starting with foundational training and team meetings, followed by regular workshops, coaching sessions, and check-ins. Activity levels peaked around mid-2021 and mid-2022, reflecting our sustained effort to support participants and ensure the program's long-term success. However, our self-critique reveals that the focus on convenience and engagement was driven by our research needs. While participants appeared engaged due to the numerous activities provided, they rarely took advantage of optional opportunities, suggesting that the engagement might have been superficial or overly structured to meet research objectives.

One additional challenge to the implementation of a community-based research identified via our contextual analysis is our ongoing ineffectiveness in reading results sociologically and collaboratively beyond their predefined scope of our research tools, such as physical assessment results, Fitbit reports, and semi-structured qualitative interviews, referred to as the inconvenience of ventriloquizing our research language.

The burden and identity effect of technical language have been critiqued in methodology studies; some argue the language must be simpler (68, 69). The language problem extends to our technical interpretations of the verbalized data used in the research process (70). The interpretive and collaborative methods throughout the intervention should prompt reflection. Listening to participant feedback during public presentations revealed a disconnect between highly technical and highly interpretive approaches.

Here is one example, when applying a contextual analysis, we notice that “competition” in relation to physical activity was not in most participants’ goal setting, which was done at the start by a research staff member. Often, in our current times, competition has opponents and proponents in the world of physical rehabilitation (71), but this is what we could have missed without a deeper analysis behind the numbers. The following quote illustrates the move towards being competitive with loved ones by a female participant. This was also reported by other participants.

My mom has a Fitbit too, and I added her as like a family member or friend. So now she gets to see how much I am doing for the day. There will be days that she’ll talk to me or whatever or like I’ll send her my badges that I’ve won… It got her more motivated to get outside and do things… I’m still always going to win just because I’m always like 40,000 steps ahead of her. It’s a good little like thing between us… My stepdad ended up getting one too just to compete with me because he says he walks as much as work as he does. So that’s also been a great little family thing. (P3; participant living with HIV; end of CBE intervention)

Our research required participants to use technical language to interpret their experiences, expecting them to adopt our specialized terminology. This practice is common in research, where participants are often expected to “ventriloquize” researchers’ expectations rather than express their own experiences. This issue, highlighted in participatory and inclusive research methodologies, suggests the need for adaptable research designs that consider participants’ natural language and responses, which may not fit neatly into predefined questions and formats (72). The hidden curriculum in health research influences participants’ understanding of the research process and their roles within it, often teaching them about expected behaviors and self-perceptions. This implicit education can impact the authenticity of participants’ responses and their engagement with the research (73).

The intervention aimed to foster motivation and increase physical activity through structured online CBE sessions. However, participants sometimes abandoned these exercises post-intervention, relying instead on family and friends for physical activity, creating self-produced schemes for friendly competition, and making adjustments to their daily routines, such as getting off transit before their stop to add extra walking. The theme of competition, which was not part of our interview guide, emerged outside our planned conversation parameters. This suggests a risk of interpreting transcripts superficially or only considering the first layer of data (74). The following excerpt shows competition as a theme in relation to goal setting, a key activity in the intervention.

Q: What, if any lessons or strategies, do you think you’ll take with you?

R: The goal setting which would be a little more specific around especially timeframes… It’s produced a side interest in nutrition and making sure that I eat better than I have been at times… And from a competitive point of view, I’m looking forward to getting back into the competitive swim circuit… I don’t think I’d have been able to do that without this maintenance period in between. (P22; participant living with HIV; end of follow-up phase)

Q: I’m guessing that you didn’t know anybody else who was in the study?

R: I do. I kind of… we created some group with a few people. There were two women and me. I’m still a man. I would like another man who can have a bit of healthy competition, you know. The age gap was different. The gender was different… We created our own WhatsApp group. The ladies talk to each other or something. But we’re from a different age group… There were some of these factors. (P14; participant living with HIV; end of CBE intervention)

Related to convenience as a metaphor, we also identified a potential burden of our well-intentioned knowledge mobilization efforts. Excessive knowledge mobilization (KM) or knowledge translation and exchange (KTE) can overwhelm health research participants, leading to information overload, increased burden, reduced authenticity, and resistance to participation. These issues can undermine intervention effectiveness, skew research findings, and decrease participant engagement (73, 75, 76).

Our knowledge mobilization efforts included six optional self-management sessions for participants, YMCA coach trainings, five conference posters, and one published manuscript. While these aimed to advance understanding, they inadvertently burdened participants and researchers. Our efforts for micro-learning and concise, engaging materials aligned with active learning and precise health communication practices were lost in a great deal of technical information. Since the early 2000s, community-based knowledge mobilization, knowledge transfer and exchange (KTE), and “knowledge bridging” have enhanced social justice by increasing “voice” and “representation” (77).

Our knowledge mobilization efforts aimed to be engaging, but the high number of activities within a short timeframe created significant pressures, especially during the COVID-19 lockdowns. This highlights the need for a more sustainable approach to maintain engagement and achieve lasting benefits. This issue is common in physical exercise pilot studies, where initial high engagement can lead to participant and researcher burnout and a decline in positive outcomes after the pilot ends (78, 79). This suggests the need for a slower, meticulous approach to public health, advocating for interdisciplinary work and a “slow movement” in research (80, 81). Localized knowledge mobilization efforts, often small-scale and community-focused, aim to shift individual perspectives using creative methods and grassroots materials (82).

The frequency of prescribed activities unfamiliar to participants, along with numerous optional knowledge mobilization activities, may contribute to “research fatigue” and disengagement. Approximately 60,000 people are formally diagnosed with HIV in Canada, and researchers frequently engage them in many research activities (83). People living with HIV must repeatedly share their stories, often under less-than-ideal conditions, despite the goal of integrated health systems, which are not yet fully realized (84).

Q: I’m wondering if there’s anything that’s influenced your access to the study or experience of the study.

R: The length of the study, it could be shortened. I think we don’t need to do it as often.

Q: You’re talking about those weekly reports?

R: Yes. Like add in a new question every now and then. Make it like this month’s weekly study is going to be… we’re going to add in nutrition and see how like you did well because it’ll also change your mindset because I can go through that questionnaire like nobody’s business… Like add in a little extra little tidbit each month to kind of “hey mental health month!”. If you guys are doing your HIV in Motion and then you’re like whatever study that we were doing, incorporate that into the weekly settings because it’ll also engage what we’re learning about with our wellness and self-care classes. (P3; participant living with HIV; end of follow-up phase)

Collectively, the elements represented by each of the four Cs emphasize the holistic approach needed to address the multifaceted challenges faced by individuals living with HIV.

## Discussion

Our findings underscore the importance of designing physical activity programs for individuals living with HIV that are affordable, emotionally supportive, non-stigmatizing, and convenient. Individuals with invisible or *invisibilized* and highly stigmatized conditions such as HIV, multiple sclerosis, and mood disorders face barriers to physical activity, including fatigue, stigma, and lack of accessible facilities, underscoring the need for inclusive, affordable, and supportive programs to enhance engagement and health outcomes (85, 86). This aligns with the four Cs framework—Cost, Care, Comfort, and Convenience—which advocates for holistic approaches to address the diverse challenges faced by this population.

**Cost** barriers, such as the costs associated with public gyms and transportation, significantly impede engagement in regular physical activity. Participants suggested that facilities such as the YMCA should re-evaluate membership fees and seek funding to support communities in need. Additionally, while basic medical services are generally accessible, physical activity was often perceived as supplementary, indicating a gap in comprehensive care. Participants also noted limited communication with healthcare providers regarding exercise, highlighting the need for integrated discussions about physical activity within clinical settings.

Reducing economic barriers for individuals with chronic, invisible, or episodic disabilities could yield significant societal benefits in resource-rich countries such as Canada and the United States. Chronic diseases alone cost the Canadian economy approximately $190 billion annually, with $122 billion attributed to income and productivity losses, suggesting that improved financial support could reduce these economic impacts (87). The Mental Health Commission of Canada also emphasizes that systemic barriers, including financial constraints, hinder socioeconomic participation, with measures such as the Canada Disability Benefit Act aimed at reducing poverty and enhancing societal engagement (88). These initiatives indicate that offering reduced costs or payment options could improve health outcomes while generating broader economic advantages.

**Care** could be improved by incorporating physical activity into treatment plans may ameliorate lingering effects of HIV that are not addressed by antiretroviral therapy and enhance the quality of life of people living with HIV (89). Additionally, integrating mobile health (mHealth) interventions, such as fitness trackers and supportive apps, has shown promise in enhancing motivation and sustaining engagement in physical activity among people living with HIV (90).

**Comfort** in this study emerged as a significant factor for individuals living with HIV, emphasizing the necessity of creating exercise environments that mitigate stigma and foster psychological ease. Participants consistently highlighted their preference for home-based, online interventions, which allowed them to avoid youth-oriented and appearance-focused public gyms. These settings often exacerbate feelings of stigma, internalized or anticipated, related to their HIV status. The importance of comfort aligns with findings that inclusive, stigma-free environments encourage engagement in physical activities for people living with HIV (46, 47).

**Convenience** encompassed factors such as time, opportunity, energy, and a sense of belonging, as well as the alleviation of enduring trauma and HIV-related stigmas. Participants reported experiencing survey fatigue due to the repetitive nature of weekly assessments and felt pressured by the numerous activities scheduled within a limited timeframe, suggesting that engagement may have been superficial or overly structured to meet research objectives.

These findings collectively highlight the importance of developing and implementing physical activity programs that are not only accessible and supportive but also tailored to the specific needs and challenges faced by individuals living with HIV. Results align with earlier work by Jiancaro et al. (16), highlighting the complex interaction of personal, environmental, social factors influencing the implementation of online CBE with adults living with HIV, and emphasizing the need for dedicated, rehabilitation-centred, and multi-skilled members to provide support online CBE.

### Strengths and limitations

Our discussion incorporates both descriptive and contextual analysis, acknowledging some overlapping results. The thematic analysis reveals optimism and positivity, but we also need to consider potential biases in community-based research. There is a potential for overestimation of the positive impact of the community-based online exercise intervention due to social desirability bias (91), and demand characteristics might have caused participants to alter responses to meet perceived expectations (92). Additionally, the participant expectancy effect could have influenced participants to report anticipated outcomes (92), and the Hawthorne effect suggests that awareness of being observed may have altered participant behavior, resulting in more positive self-reporting (93). These biases indicate that a superficial thematic analysis revealing optimism and positivity might be inflated due to these psychological and social influences on the participants’ responses. While we achieved our targeted sample size of recruiting 10 adults living with HIV and five CBE representatives to the study, not all participants completed all three interviews. As a result, we recruited an additional three adults living with HIV and three CBE representatives to the study. This resulted in a total of 30 interviews with adults living with HIV and 13 interviews with CBE representatives, specifically 15 interviews at initiation (month 0), 12 interviews at the end of CBE intervention (month 6), and 16 interviews at the end of follow-up (month 12) (Tables 1, 2). The majority of participants living with HIV completed all three interviews (*n* = 7), and while our aim was not to achieve saturation in this study, we received sufficient data to address our study objectives, exploring experiences with CBE implementation. Nevertheless, results are limited to participants who remained engaged in the online CBE intervention, and may not be transferable to the broader population of adults living with HIV.

## Conclusions

This study highlights the critical importance of designing physical activity interventions that are affordable, emotionally supportive, non-stigmatizing, and convenient for individuals living with HIV. The “four Cs” framework—Cost, Care, Comfort, and Convenience—emerged as a guiding metaphor for addressing the multifaceted challenges faced by this population. Economic barriers, such as the costs of transportation and gym memberships, coupled with limited discussions about exercise in healthcare settings, underscore the need for integrated, inclusive solutions. Participants living with HIV preferences for home-based online interventions underscore the importance of comfort and reducing stigma in physical activity settings. Additionally, the challenges of maintaining engagement, such as assessment fatigue and technological barriers, illustrate the importance of tailoring programs to participants’ needs while minimizing their burdens. Future efforts must integrate interdisciplinary approaches, embrace community-based principles, and prioritize participant-centered designs to ensure sustainable health outcomes and advance the implementation of effective HIV-related interventions. Findings are limited to adults living with HIV who remained engaged in the online CBE intervention. Future research should involve broader scale-up and implementation with a larger sample of persons living with HIV.

## Data Availability

Results of the study are supported by the data with supportive quotes in this article. The complete dataset is available upon reasonable request from the corresponding author. Requests to access the datasets should be directed to KO’B, kelly.obrien@utoronto.ca.
