# Supplementary material for: Qualitative insights from an online community-based exercise intervention for persons living with HIV
Source: Front Rehabil Sci. 2025 Aug 25;6:1602007. doi: 10.3389/fresc.2025.1602007 (PMC12415039; doi:10.3389/fresc.2025.1602007)
Supplement: Supplementary file 1 [file Datasheet1.pdf]

INTERVIEW GUIDE

ADULTS LIVING WITH HIV

Thank you for agreeing to participate in this study. I am meeting with you over the course of this study, to try to get a better understanding of your experience within the Tele-Coaching CBE Intervention, specifically your thoughts on any changes in your health *outcomes* as well as the *process (Implementation & Adoption)* of engaging in Tele-Coaching CBE. This part of the study involves participating in 3 interviews over the course of the Tele-Coaching CBE Intervention Study.

Overview of Interviews

Initiation of the CBE Intervention Interview: In this first interview at baseline, just before you start the CBE intervention, we would like to get a sense of a) your *experience* with exercise to date, b) any *goals or anticipated benefits* of participating in the upcoming CBE intervention, and c) any *concerns or perceived challenges* that you might have leading up to the start of the CBE intervention.

After CBE Intervention Interview: The second interview will occur when after you complete the exercise intervention. In this interview, we would like to get a better understanding of a) the *process* of participating in the Tele-coaching CBE intervention, b) any *perceived changes* (either benefits or harms) of the Tele-Coaching CBE intervention on your health and disability and your engagement in the care cascade since you started the exercise program, c) strengths and challenges taking part in the Tele coaching CBE intervention, and c) your intention to continue to *engage* in the CBE post-intervention. We are specifically interested in your experiences, strengths, challenges of use of the technology for the intervention.

After the Follow-Up Maintenance Phase Interview (End of Study): The third and final interview will occur after you complete the entire study. In this interview, we would like to obtain your thoughts on the overall a) *process* (strengths and challenges) of participating in the CBE st, b) any further updates on the *perceived impact* (benefits or harms) of Tele-Coaching CBE on your health and engagement in the care cascade over time, and c) your *intention* to continue to engage in CBE post study. Again, we are interested in your experiences, strengths, challenges of use of the technology for the intervention.

In addition, in the second and third interviews, we will specifically explore the *influence of extrinsic factors* such as social support or stigma and *intrinsic factors* such as concurrent health conditions, gender, age and coping strategies [contextual factors in the *Episodic Disability Framework]* on the impact and your level of engagement or adherence with exercise. We will also ask you about your experience and ability of using the intervention online (tele-coaching) and the technology. We are interested in learning about what you think makes it easier or harder to access and participate in tele-coaching community based exercise and whether there should be any refinements made to this Tele-Coaching CBE intervention before more broadly implementing with the community. Results from these interviews will be used for the future refinement of an accessible, feasible, safe, and sustainable Tele-Coaching CBE program for people living with HIV. Before we start, do you have any questions?

INTERVIEW #1 - BASELINE (INITIATION OF CBE) INQURY – People Living with HIV Participants

PERSONAL DOMAIN: EXPERIENCE WITH EXERCISE / PHYSICAL ACTIVITY [NEW SUBSECTION]

1) To begin, can you tell me what brought you to this program?
2) Can you tell me about your **past experience with exercise or physical activity**? [NEW]
    a. Were you involved in training, sports, or other kinds of physical activity in the past?
    b. If so, can you say more about that
        i. Type, frequency, duration, intensity (mild, moderate, intense)?
        ii. When? (before the pandemic?) Where (at home?)
        iii. Equipment/Technology? Cost?
    c. If you stopped with any of these activities, what would you say caused you to stop?

3) What is your ***current experience with exercise / physical activity***?
    a. Are you currently engaging in regular exercise?
    b. If so, can you describe the type, frequency, duration, intensity (mild, moderate, intense)?
    c. Where (home?) Equipment / technology?
    d. Do you have any kind of exercise equipment now (besides what's part of the program)?

PERSONAL DOMAIN: CBE PROGRAM…. PREFERENCES, VALUES [NEW SUBSECTION]

3) As you know, this program is online, but given the choice, would you choose an online program like this one or an in-person program? [NEW]
    a. Why would that be your preference?

4) Can you talk about what attracts you to this program? [NEW]

5) Along a similar line, do you ***anticipate any benefits*** from participating in this program? [RE-WORDED FOR ME]
    a. If so what might they be?
        i. Improvements in health or reduction of disability? (mental health, physical health, social inclusion, uncertainty, cognitive health)
        ii. Increased social interaction with others
        iii. Reduced feelings of stigma (specifically with ability to exercise at home).

6) Do you have any ***concerns or ~~perceived~~ foresee any challenges*** in participating in the program? [RE-WORDED / RE-ORDERED]

    Concerns with …

    a. Exercising at home
    b. Keeping up with the exercise
    c. Attending regularly

d. Feelings of stigma
e. Potential for episodes of illness (or other life triggers that may arise)
f. Engaging long term with in an exercise program (over time)
g. With privacy of exercising in the home
[SEGUE TO NEXT QUESTION]

TECHNOLOGY DOMAIN [NEW SUBSECTION]

h. Ability to use the tele-coaching technology [e.g. laptop, apps]
i. Fitbit
j. Concerns with technology

7) How do you ***feel about adopting new technology*** necessary for the program?
    a. Concerns with using the technology (use, confidentiality, connection etc.)
    b. Comfort level using the technology
    c. Willingness to learn to use the technology
    d. Confidence in using the technology
8) Continuing on the topic of technology, do you foresee any **technical difficulties** while you're enrolled in the program? [internet strength, device problems, application problems]
9) Generally, do you find technical issues to be an obstacle or is it something you typically find manageable?

HOME DOMAIN [NEW SUBSECTION]

4) Would you say that there's a place in your home that's suitable for exercise? [space, quiet, private]
5) Can you describe this space? {e.g. suited to doing cardio, to floor exercises or stretches)
6) Is it also outfitted with suitable technology [e.g. laptop] to go online?

PERSONAL DOMAIN: GOALS, MOTIVATIONS, MEANING [NEW SUBSECTION]

10) Have you given any thought to the ***goals*** you have for the program [RE-WORDED]?
    a. If so, what are they?
        i. Vague or specific goals?
        ii. Physical health goals? Mental health goals?
    b. In the big picture, what is it about this goal that's important/valuable to you [MOTIVATION / MEANING]
    c. Can you say more on this? (What inspires you? How do you think you might best achieve this goal?)

11) Some people experience **health-related challenges associated with HIV** (physical, social, mental or cognitive) Are you currently ***living with these kinds of health related challenges?*** If so, can you describe what they are? [RE-WORDED FOR TJ - interviewer]

12) These are all the questions I planned. Is there anything you'd like to add? [NEW]

INTERVIEW #2 – POST CBE INTERVENTION LINE OF INQUIRY – People Living with HIV Participants
Perceived Impact of Exercise from perspective of participants

Can you describe your ***experience*** so far participating in the Tele-Coaching CBE intervention?

a. How did you come to access **the study** (or start up)?
b. Why did you sign up for the study? What was appealing about it?
   i. **CBE Intervention – One-on-one Coaching**
      - Can you tell me what it is like to be involved in the one-on-one coaching as part of the of the CBE intervention?
   ii. ***Technology – Tele-coaching - CBE Intervention***
      - Can you tell me what it was like being coached by your fitness instructor online?
   iii. **CBE Intervention – Exercise Program**
      - Can you tell me what it was like engaging in exercise at your house?
      - Have you accessed the online group classes via Sweat for Good App?
      - Have you been exercising at the YMCA (if open)? Or exclusively at home?
   iv. **Self-Management Sessions (online or in person)**
      - Did you attend the self-management sessions online or in person?
      - Can you tell me what it is like to be involved in the self-management sessions with the CBE intervention?
   v. **Fitbit Inspire 2**
      - Did you regularly wear your Fitbit Inspire 2?
      - Did you use your Fibit Inspire 2 as a way to track your physical activity?
      - Did you find wearing your Fitbit Inspire 2 motivated you to exercise?
c. What has it been accessing the **fitness coaching sessions online at home**?
d. What ***types of activities*** are you engaging in?
e. Does the ***individual nature of the coaching sessions*** make a difference (one-on-one engagement)? If so, how?
f. What has your ***attendance*** (or adherence) been like so far with the CBE intervention (online tele-coaching sessions, 3x weekly exercise, self-management sessions, Fitbit Inspire 2)? Are you attending your coaching sessions/YMCA/Exercising at home regularly? If not, why not? If yes, what are some of the reasons you're attending regularly?

Fidelity (adherence; dose or amount of intervention delivered; and quality of delivery (e.g. tele-coaching).

2) Can you walk through and describe **your most recent coaching session** from start to finish?
3) Can you walk through and explain the events of **your most recent independent exercise session** from start to finish?

Group versus Individualized Sessions

g. Were you engaged in any online 'group-based activities' for your exercise program apart from your coaching sessions?

i. Have you met / interacted with any other CBE participants? If so, can you describe? (For instance, are you running into other CBE participants at the YMCA (if applicable)?

j.1. Did you know them before the study, or did you meet them as part of your involvement in the study?).

j.2. Can you describe your exchanges (if any) with the other participants in the study?

j. Overall, can you comment on the extent to which you feel there is potentially a 'sense of community' developed (or not) with your peer and other CBE participants? If so, how? What has changed?

4) Have you noticed ***any changes in your health or disability (e.g. benefits or harms)*** since starting the intervention? If so, what were they? Overall, what do you think the ***impact of the tele-coaching CBE intervention*** has been for you thus far?

Probe: When we met last time, you mentioned [*REFER TO BASELINE TRANSCRIPT*], has anything changed since then? If so, how has it changed?

a. Physical health
b. Mental health
c. Cognitive health
d. Social inclusion
e. Daily functional activities
f. Uncertainty or worrying about the future

5) Have there been any ***environmental factors*** that have influenced your experience with the tele-coaching CBE intervention? If so, can you describe? How might they influence the impact of exercise on your health?

a. Social support (support from friends and family to engage in exercise; support from health providers; fitness instructors)
b. Stigma (reduction of stigma engaging in tele-coaching or exercising in group with other PHAs)
c. Feasibility of technology using online tele-coaching
d. Interactions with coach (Are they progressing the exercises? Are they correcting your form during the exercises? Did you feel motivated to see your coach each session?)
e. Interactions with research staff
f. Wearing a Fitbit

6) Have there been any ***personal factors*** that have influenced your experience with the tele-coaching CBE intervention? (either your ability to participate in the exercise; or the changes you might see with your health)? If so, can you describe? How might they influence the impact of exercise on your health?

a. Personal attributes (age, gender, ethnocultural background, length of time since diagnosis)
    i. How has your ***gender*** influenced your ability to engage in exercise?
b. Influence of other concurrent health conditions
c. Ability to cope with HIV, living strategies (nutrition, medication adherence, smoking, etc)

Tele-Coaching Technology Experiences

7) How would you ***describe your experience using the technology*** as part of the CBE Tele-coaching intervention?
    a. Usability and ease of usage of technology
        i. Confidence in use
        ii. Comfort level
        iii. Ease of navigation
        iv. Learnability of the technology over time
    b. Satisfaction with technology
        i. Quality (visual, sound etc.)
        ii. Effectiveness of delivering coaching sessions
        iii. Engagement with coach through technology
        iv. Ability to understand coaches instructions through the technology
    c. Reliability of the technology
        i. Online technology connection
        ii. Confidence in technology to deliver your coaching session
        iii. Interruptions
        iv. Responsiveness of technology (was there any technological delays or pauses?)
        v. Confidentiality or privacy concerns
    d. Feasibility
        i. Would you continue to use technology for fitness coaching in the future? Why or why not?

8) Did you ***access the online YMCA group classes via the Sweat for Good App***? If yes, how was your experience with the class through this technology?
    a. Usability and ease of usage of online classes
    b. Satisfaction with the online classes (visual and sound quality)
    c. Reliability of the technology (connection, interruptions, responsiveness)
    d. Confidentiality of the online classes
    e. Feasibility of the online classes
    f. Future online class use

Process of CBE Translation – Including Adoption and Implementation

Questions adapted for RE-AIM Planning Tool - series of 'thought questions' which serve as a checklist for key issues to consider when evaluating the intervention.

9) Have there been any ***strengths or challenges*** associated with the translation of the tele-coaching CBE intervention into the HIV community? (e.g. What aspects of the CBE intervention do you like? Not like?)
    a. Online tele-coaching technology (feasibility, use, comfort, satisfaction)
    b. Online fitness instructors
    c. Timing of the exercise sessions
    d. Online exercise classes
    e. Accessibility of exercising on your own at home
    f. Monthly self-management education sessions
    g. Equipment (Fitbit)
    h. Engaging with other participants
    i. Stigma
    j. Ability to keep up with the sessions
    k. Ability to attend the sessions regularly
    l. Experiences of episodic illness as a trigger that might influence ability to exercise

10) What are your thoughts on your ***ability and willingness to continue to engage in exercise over time?***
    a. Do you plan to continue with the exercise in the next phase? If yes, why? If not, why not?
    b. ***When we met last time, you mentioned [REFER TO BASELINE TRANSCRIPT], has anything changed since then?*** **If so, how has it changed?**

11) How confident are you that you will be ***able to adopt exercise*** into part of your lifestyle over the long term? Can you describe in more detail?
    a. ***When we met last time, you mentioned*** **[*REFER TO BASELINE TRANSCRIPT*]*, has anything changed since then?*** **If so, how has it changed?**

12) How confident are you that the intervention (or program) can be ***consistently delivered*** (or implemented) as it is intended?
    a. What might be the greatest threat **(or challenge)** to consistently implementing a tele-coaching CBE program in the community? Do you have any ideas on how to overcome these barriers?

13) How confident are you that other community-based organizations (such as other YMCAs or other community organizations) may be ***willing and able to offer*** this tele-coaching intervention as a fully developed program?
    a. What do you think will be the greatest barriers to other sites or organizations adopting this tele-coaching intervention? Do you have any ideas on how to overcome these barriers?

14) Do you have any suggestions for ways in which to ***sustain the tele-coaching CBE program*** over the long term in the HIV community?

15) Do you have suggestions on ***other partnerships or stakeholders*** that should be considered in the long term sustainability of a tele-coaching CBE program? If so, who are they?

INTERVIEW #3 – POST STUDY LINE OF INQUIRY – People Living with HIV Participants

[similar to midpoint inquiry with questions about long term sustainability]

Perceived Impact of Exercise from perspective of PLWH participants

16) Can you describe your ***experience*** so far participating in the Tele-Coaching CBE intervention?
    - i. **CBE Intervention – One-on-one Coaching**
        - Can you tell me what it is like now that you no longer have the one-on-one coaching as part of the of the CBE intervention?
    - *ii.* ***CBE Intervention – Tele-Coaching***
        - Can you tell me what it was like being coached by your fitness instructor online?
    - iii. **CBE Intervention – Exercise Program**
        - Can you tell me what it was like engaging in exercise at your house?
        - Have you accessed the YMCA online group classes?
        - Have you been exercising at the YMCA? Or exclusively at home?
    - iv. **Self-Management Sessions (online or in person)**
        - Did you attend the self-management sessions online?
        - Can you tell me what it is like to be involved in the self-management sessions with the CBE intervention?
    - v. **Fitbit Inspire**
        - Did you regularly wear your Fitbit Inspire?
        - Did you use your Fitbit Inspire as a way to <u>track</u> your physical activity?
        - Did you find wearing your Fitbit Inspire <u>motivated</u> you to exercise?

    b. How has it been **exercising at home** (without an online coach)?
    c. Does the **individual nature of the exercise sessions** (without one-on-one coaching) make a difference?
    d. What ***types of activities*** are you engaging in?
    e. What has your ***attendance*** (or adherence) been like so far with the CBE intervention (3x weekly exercise, Fitbit Inspire)? Are you exercising at home regularly? If not, why not? If yes, what are some of the reasons you're attending regularly?

Fidelity (adherence; dose or amount of intervention delivered; and quality of delivery (e.g. tele-coaching).

17) Can you walk through and describe <u>**your most recent exercise (either a group class or independent) session**</u> from <u>start to finish</u>?

Group versus Individualized Sessions

    f. Are you engaged in any '***group-based activities'*** for your exercise program?

g. Have you ***met / interacted with any other CBE participants***? If so, can you describe? (For instance, are you running into other CBE participants at the YMCA?
    f.1. Did you know them before the study, or did you meet them as part of your involvement in the study?).
    f.2. Can you describe your exchanges (if any) with the other participants in the study?
h. How has the coordinated peer support been going? Do you connect with your peer regularly?
i. – Overall, can you comment on the extent to which you feel there is potentially a '***sense of community'*** developed (or not) with other CBE participants? If so, how? What has changed?

18) Have you noticed ***any changes in your health or disability (e.g. benefits)*** after participating in the tele-coaching CBE intervention? If so, what were they (Improvements? Deterioration)? Overall, what do you think the ***impact of the tele-coaching CBE intervention*** has been for you thus far?

Probe: When we met last time, you mentioned [*REFER TO BASELINE TRANSCRIPT*], has anything changed since then? If so, how has it changed?

Specific probes:

a. Physical health
b. Mental health
c. Cognitive health
d. Social inclusion
e. Daily functional activities
f. Uncertainty and worrying about the future

19) Have there been any ***environmental factors*** that have influenced your experience with the tele-coaching CBE intervention? If so, can you describe? How might they influence the impact of exercise on your health?

j. Social support (support from friends and family to engage in exercise; support from health providers; fitness instructors)
k. Stigma (reduction of stigma engaging in tele-coaching or exercising in group with other PHAs)
l. Feasibility of technology using online tele-coaching
m. Interactions with coach (Are they progressing the exercises? Are they correcting your form during the exercises? Did you feel motivated to see your coach each session?)
n. Interactions with research staff
o. Wearing a Fitbit

20) Have there been any ***personal factors*** that have influenced your experience with the tele-coaching CBE intervention? (either your ability to participate in the exercise; or the changes you might see with your health)? If so, can you describe? How might they influence the impact of exercise on your health?

Probe: When we met last time, you mentioned [*REFER TO BASELINE TRANSCRIPT*], has anything changed since then? If so, how has it changed?

a. Personal attributes (age, gender, ethno-cultural background, length of time since diagnosis)

        i. How has your **gender** influenced your ability to engage in exercise?

   b. Living with concurrent health conditions
   c. Ability to manage health challenges with HIV, living strategies (nutrition, medication adherence, smoking, etc)

Tele-Coaching Technology Experiences

21) Did you ***continue to receive online tele-coaching sessions*** during the follow-up phase (out of pocket)?

22) a) If no, ***would you consider using online tele-coaching*** in the future?

    b) If yes, how would you ***describe your experience using the technology*** as part of the CBE Tele-coaching intervention?

   a. Usability and ease of usage of technology
      i. Confidence in use
      ii. Comfort level
      iii. Ease of navigation
      iv. Learnability of the technology over time
   b. Satisfaction with technology
      i. Quality (visual, sound etc.)
      ii. Effectiveness of delivering coaching sessions
      iii. Engagement with coach through technology
      iv. Ability to understand coaches instructions through the technology
   c. Reliability of the technology
      i. Online technology connection
      ii. Confidence in technology to deliver your coaching session
      iii. Interruptions
      iv. Responsiveness of technology (was there any technological delays or pauses?)
      v. Confidentiality or privacy concerns
   d. Feasibility
      i. Would you continue to use technology for fitness coaching in the future? Why or why not?

23) Did you ***access the online YMCA group classes***? If yes, how was your experience with the class through this technology?
   a. Usability and ease of usage of online classes
   b. Satisfaction with the online classes (visual and sound quality)
   c. Reliability of the technology (connection, interruptions, responsiveness)
   d. Confidentiality of the online classes
   e. Feasibility of the online classes
   f. Future online class use

Process of CBE Translation – Including Adoption and Implementation

Questions adapted for RE-AIM Planning Tool - series of 'thought questions' which serve as a checklist for key issues to consider when evaluating the intervention.

24) Have there been any ***strengths or challenges*** associated with the translation of the tele-coaching CBE intervention into the HIV community? (e.g. What aspects of the CBE intervention do you like? Not like?)
    m. Online tele-coaching technology
    n. Online fitness instructors
    o. Timing of the exercise sessions
    p. Online exercise classes
    q. Accessibility of exercising on your own at home
    r. Monthly self-management education sessions
    s. Equipment (Fitbit)
    t. Engaging with other participants
    u. Stigma
    v. Ability to keep up with the sessions
    w. Ability to attend the sessions regularly
    x. Experiences of episodic illness as a trigger that might influence ability to exercise

25) What are your thoughts on your ***ability and willingness to continue to engage in exercise over time?***
    a. Do you plan to continue with the exercise in after the study is completed? If yes, why? If not, why not?

26) How confident are you that you will be ***able to adopt exercise*** into part of your lifestyle over the long term? Can you describe in more detail?

27) How confident are you that the intervention (or program) can be ***consistently delivered*** (or implemented) as it is intended?
    a. What might be the greatest threat to consistently implementing a tele-coaching CBE program in the community? Do you have any ideas on how to overcome these barriers?

28) How confident are you that other community-based organizations (other YMCAs or other community organizations) may be ***willing and able to offer*** this tele-coaching intervention as a fully developed program?
    a. What do you think will be the greatest barriers to other sites or organizations adopting this intervention? Do you have any ideas on how to overcome these barriers?

29) Do you have any suggestions for ways in which to ***sustain the tele-coaching CBE program*** over the long term in the HIV community?

30) Do you have suggestions on ***other partnerships or stakeholders*** that should be considered in the long term sustainability of a tele-coaching CBE program? If so, who are they?

Summary

Is there anything else you would like to say about participation the CBE intervention?

Thank you very much for participating in this interview today. Your responses will help to provide a better understanding of the perceptions of CBE programs among people living with HIV and will help to formulate a future program down the road.

INTERVIEW GUIDE

Representatives Involved in CBE Implementation

[Process (strengths and challenges) of translating the CBE Intervention] and Feasibility of Sustainability

Thank you for agreeing to participate in this study. I am meeting with you over the course of this study, to try to get a better understanding of your experience and/or perspectives on implementing a tele-coaching CBE Intervention, specifically *process* and *feasibility* of implementing the tele-coaching CBE intervention with PLWH. This part of the study involves participating in 3 interviews over the course of the tele-coaching CBE Intervention Study.

Overview of Interviews

Initiation of CBE Intervention Interview: In this first interview at baseline just before you start to implement the CBE intervention, we would like to get a sense of any anticipated strengths, concerns or perceived challenges that you might have leading up to the initiation of the CBE intervention.

After CBE Intervention Interview: The second interview will occur, following completion of the intervention. In this interview, we would like to obtain an understanding of things are going thus far, specifically a) how the *process* of translating the CBE intervention is going to date.

After Follow-Up Maintenance Phase (End of Study) Interview: The third and final interview will occur following completion of the study. In this interview, we would like to obtain your thoughts on your overall a) *process* (strengths and challenges) of translating the tele-caching CBE intervention, and b) the *feasibility of sustaining* the tele-coaching CBE intervention with the YMCA over the long term. Before we start, do you have any questions?

INTERVIEW #1 - BASELINE LINE OF INQURY – Representatives Involved in CBE Implementation

INTRO

- Overview: Complex intervention. Interest in discussing different aspects of the intervention: People (community, organizational), Profits (business) & Technological Parts (online tech) that comprise it [NEW]
- Topics: strengths, concerns, challenges, and processes
- Including: Feasibility, Sustainability with respect to the different aspects
- Time frame: Now and in future
- *If necessary, the interviewer will describe the intervention [NEW}

INTERVIEWEE BACKGROUND [NEW HEADING]

* Can you describe your organization and your role? [NEW]

1 Your experience implementing … [programs like this one]

What is your ***experience with implementing CBE exercise programs?*** Any experience working specifically with adults living with **HIV** or members of the HIV community?

PEOPLE ASPECT: COMMUNITY ASPECTS (PLWH) …. [NEW HEADING]

3 Perceived benefits of a program like this … From your perspective …

What are some of the perceived ***benefits for people living with HIV*** who participate in the tele-coaching CBE Intervention?

a. Improvements in health or reduction of disability? (**mental/ cognitive/ social/ physical domains** of health)
b. Increased social interactions with others (Did you notice CBE participants interacting with each other? Can you describe the types of interactions? Do you encourage participants to engage with each other in any exercise activities?
c. Do you feel that engagement in the CBE study has changed feelings of **stigma** in any way? (stigma on the part of other recreation providers, feelings of stigma and discrimination among the participants?)

ORGANIZATIONAL ASPECTS [NEW HEADING]

*[For YMCA staff] How would you describe your experience during launch with the implementation team? [NEW]

*[**For other stakeholders**] Thinking about organizational aspects, what has your experience been launching programs like this? [NEW]

- Communication. Level of support. Training. Admin.
- Understanding of HIV/Health conditions

4 What might be some of the ***benefits*** [for you / to your org.] … in translating [a program like this / this intervention] to the community?

a. Increased membership
b. Increased profile of YMCA in the HIV community
c. Evaluation of a tele-coaching intervention

2 Do you / does your organization (e.g. YMCA) have any ***goals*** for translating [a program like this / this intervention] the tele-coaching CBE intervention more broadly to the HIV community?

a. If so, what are they?
b. How do you think you might best achieve them?
c. What do you think the benefits of this CBE intervention are for PHAs? For the YMCA?

5 Long-term practicality / **feasibility** of this program

.. Again, Thinking about translating this intervention more broadly to the community…

Assuming there's evidence in favour of a program like this, do foresee concerns or challenges translating this intervention to the community? [NEW]

Do you have any ***concerns or perceived challenges*** translating the tele-coaching CBE intervention to the community?

a. Evidence. Maintaining up-to-date on the evidence on HIV and exercise
b. Staffing. Full-time versus part-time YMCA staff/coach differences at all?
c. WFH: Issues surrounding trainers working from home [NEW]
d. Ethics. Issues around confidentiality

FINANCIAL ASPECTS [NEW HEADING]

e. Costs to operationalize the intervention (probes: time/ equipment/technology/ scheduling/ administrative tasks/ manpower)
f. Long term feasibility [practicality] and sustainability [maintainability] over time

Still on the topic of scaling up, …

*Do you foresee any other challenges … in translating this intervention to the community? (e.g. safety, security, legal, ethical, other) [NEW]

PERSONAL ASPECTS AS A [INSERT ROLE] ……. [NEW HEADING]

*As a [role], do you have any personal goals / priorities related to [interventions like this / this intervention]? [NEW]

*Do you perceive challenges you face as a [role] in achieving this goal? [NEW]

TELE-COACHING TECHNOLOGY ASPECTS

6 What are your **thoughts on an exercise intervention** being implemented online?

1) Participants ability to use the technology
2) Satisfaction with technology
    a. Quality (visual, sound etc.)
    b. Effectiveness of delivering coaching sessions
    c. Coaching engagement through technology
    d. Ability to understand coaches instructions through the technology
3) Reliability of the technology
    a. Online technology connection
    b. Confidence in technology to deliver coaching sessions
    c. Interruptions
    d. Responsiveness of technology
    e. Confidentiality or privacy concerns
4) Technological Feasibility
    a. Concerns with implementation of technology for online coaching sessions and group classes.
5) Technological Sustainability of tele-coaching and online classes following completion of the study

CLOSING [NEW HEADING]

*Anything to add? [NEW]

2) What is your ***experiences with implementing CBE exercise programs?*** Any experience working specifically with adults living with HIV or members of the HIV community?

3) Does your organization (e.g. YMCA) have any ***goals*** for translating the tele-coaching CBE intervention with the HIV community?
    a. If so, what are they?
    b. How do you think you might best achieve them?
    c. What do you think the benefits of this CBE intervention are for PHAs? For the YMCA?

4) What are some of the perceived ***benefits to people living with HIV*** participating in the tele-coaching CBE Intervention?
    a. Improvements in health or reduction of disability? (mental/ cognitive/ social/ physical domains of health)
    b. Increased social interactions with others (Did you notice CBE participants interacting with each other? Can you describe the types of interactions? Do you encourage participants to engage with each other in any exercise activities?
    c. Do you feel that engagement in the CBE study has changed feelings of stigma in any way? (stigma on the part of other recreation providers, feelings of stigma and discrimination among the participants?)

5) What might be some of the ***benefits to*** translating this tele-coaching CBE intervention with the community?
    a. Increased membership
    b. Increased profile of YMCA in the HIV community
    c. Evaluation of a tele-coaching intervention

6) Do you have any ***concerns or perceived challenges*** translating the tele-coaching CBE intervention with the community?
    a. Long term feasibility and sustainability over time
    b. Costs to operationalize the intervention (probes: time/ equipment/technology/ scheduling/ administrative tasks/ manpower)
    c. Maintaining up to date on the evidence on HIV and exercise
    d. Full-time versus part-time YMCA staff/coach differences at all?
    e. Issues around confidentiality

Tele-Coaching Technology

7) What are your **thoughts on the exercise intervention** being implemented through tele-coaching technology?
    a. Participants ability to use the technology
    b. Satisfaction with technology
        i. Quality (visual, sound etc.)
        ii. Effectiveness of delivering coaching sessions
        iii. Coaching engagement through technology
        iv. Ability to understand coaches instructions through the technology

c. Reliability of the technology
    i. Online technology connection
    ii. Confidence in technology to deliver coaching sessions
    iii. Interruptions
    iv. Responsiveness of technology
    v. Confidentiality or privacy concerns
d. Feasibility
    i. Concerns with implementation of technology for online coaching sessions and group classes.
e. Sustainability of tele-coaching and online classes following completion of the study

----

INTERVIEW #2 – POST INTERVENTION INTERVIEW LINE OF INQUIRY – Representatives Involved in CBE Implementation

Process of CBE Translation – Including Adoption and Implementation

Questions adapted for RE-AIM Planning Tool - series of 'thought questions' which serve as a checklist for key issues to consider when evaluating the intervention.

1) Can you describe your ***experience*** so far translating in the tele-coaching CBE intervention with the HIV community?
    a. What specifically was your role in the tele-coaching CBE intervention?
    b. What have been the good things thus far?
    c. What have been some of the challenges?
        i. In-Person Coaching
            1. Strengths and challenges
    d. What are some of your perceptions on the impact of the intervention to participants?
    e. What do you think about the tele-coaching CBE intervention, overall? Were you able to meet up with each participant every two weeks, as planned out?
    f. Can you describe the extent to which the pre-intervention knowledge exchange workshops were helpful (or not) in preparing you for delivering the CBE?
    g. Did you find the tele-coaching CBE intervention study protocol was clear? If not, do you have any suggestions on ways the communication could be strengthened?

Fidelity (adherence; dose or amount of intervention delivered; and quality of delivery (e.g. tele-coaching).

2) Can you walk through and describe **your most recent coaching session** with a participant from start to finish?

3) What are some of the ***strengths or challenges*** associated with the translation of the tele-coaching CBE intervention into the HIV community?
    a. People living with HIV accessing the program
    b. Fitness instructors
    c. Exercise testing – scheduling, costs, etc.

d. Online nature of the coaching sessions
e. Technology (feasibility, comfort, use, satisfaction)
f. Timing of the exercise sessions
g. Monthly self-management education sessions
h. Equipment availability, knowledge of how to use, etc.
i. Fitbit
j. Participants exercising at home (lack of equipment)
k. Accessibility of the environment
l. Participants engaging with other participants
m. Ability for participants to attend the sessions regularly
n. Logistics to coordinate space and equipment (at home vs at the YMCA)
o. Experiences of episodic illness as a trigger that might influence ability to exercise

4) What are your thoughts on the ***ability and willingness of people living with HIV to continue to engage in exercise over time?***

5) How confident are you that the YMCA will be ***able to adopt the tele-coaching CBE*** over the long term (sustain the program over time)?

6) How confident are you that the CBE program can be ***consistently delivered*** (or implemented) as it is intended over time?
   a. What might be the greatest **challenge (or** threat) to consistently implementing a tele-coaching CBE program in the community? Do you have any ideas on how to overcome these barriers?
   b. How did the process of accomplishing each month's tasks work for you? (probe for ease and difficulty)
   c. What are your thoughts on the format of the tele-coaching CBE intervention for people living with HIV? (i.e. bi-weekly online coaching sessions, working out at home, receiving coaching at home, exercising x3/week for an hour etc.)

7) How confident are you that other community-based organizations (other YMCAs or other community organizations) may be ***willing and able to offer*** this tele-coaching intervention as a fully developed program?
   a. What do you think will be the greatest barriers to other sites or organizations adopting this intervention? Do you have any ideas on how to overcome these barriers?

8) Do you have any suggestions for ways in which to ***sustain the tele-coaching CBE program*** over the long term in the HIV community?

9) How ***useful*** do you think a tele-coaching CBE intervention such as this one is for people living with HIV?

10) Do you have any suggestions for ways in which to ***continue the tele-coaching CBE program*** over the long term in the HIV community?

11) Do you have suggestions on ***other partnerships or stakeholders*** that should be considered in the long term sustainability of a tele-coaching CBE program? If so, who are they?

Tele-coaching Technology

12) ***a) CBE Coaches:*** What was your ***experience coaching participants through tele-coaching***?
b) Other CBE Stakeholders: What are your **thoughts on the exercise intervention** being implemented through tele-coaching technology?
    a. Participants ability to use the technology
    b. Satisfaction with technology
        i. Quality (visual, sound etc.)
        ii. Effectiveness of delivering coaching sessions
        iii. Coaching engagement through technology
        iv. Ability to understand coaches instructions through the technology
    c. Reliability of the technology
        i. Online technology connection
        ii. Confidence in technology to deliver coaching sessions
        iii. Interruptions
        iv. Responsiveness of technology
        v. Confidentiality or privacy concerns
    d. Feasibility
        i. Concerns with implementation of technology for online coaching sessions and group classes.
    e. Sustainability of tele-coaching and online classes following completion of the study

---

INTERVIEW #3 - POST STUDY LINE OF INQUIRY – Representatives Involved in CBE Implementation

[similar to midpoint inquiry with questions about long term sustainability]

Process of CBE Translation – Including Adoption and Implementation

Questions adapted for RE-AIM Planning Tool - series of 'thought questions' which serve as a checklist for key issues to consider when evaluating the intervention.

1) Can you describe your overall ***experience*** translating in the CBE intervention with the HIV community?
    a. What was your role in the CBE tele-coaching intervention?
    b. What have been the good things thus far?
    c. What have been some of the challenges?
        i. In-Person Coaching Sessions
            1. Strengths and Challenges
    d. What are some of your perceptions on the impact of the intervention to participants?
    e. What do you think about the tele-coaching CBE intervention, overall? Were you able to meet up with each participant once every 2 weeks, as planned out?

f. Can you describe the extent to which the pre-intervention knowledge exchange workshops were helpful (or not) in preparing you for delivering the CBE?

2) Did you find the tele-coaching CBE intervention study protocol was clear? If not, do you have any suggestions on ways the communication could be strengthened?
What are some of the ***strengths or challenges*** associated with the translation of the tele-coaching CBE intervention into the HIV community?
    a. PHAs accessing the program
    b. Fitness instructors
    c. Exercise testing – scheduling, costs, etc.
    d. Online nature of the coaching sessions
    e. Technology
    f. Timing of the exercise sessions
    g. Monthly self-management education sessions
    h. Equipment availability, knowledge of how to use, etc.
    i. Participants exercising at home (lack of equipment)
    j. Accessibility of the environment
    k. Participants engaging with other participants
    l. Ability for participants to attend the sessions regularly
    m. Logistics to coordinate space and equipment (at home vs at the YMCA)
    n. Experiences of episodic illness as a trigger that might influence ability to exercise

3) What are your thoughts on **the *ability and willingness of people living with HIV to continue to engage in exercise over time?***

4) How confident are you that the YMCA will be ***able to adopt the tele-coaching CBE*** over the long term (sustain the program over time)?

5) How confident are you that the CBE program can be ***consistently delivered*** (or implemented) as it is intended over time?
    d. What might be the greatest **challenge (or** threat) to consistently implementing a tele-coaching CBE program in the community? Do you have any ideas on how to overcome these barriers?
    e. How did the process of accomplishing each month's tasks work for you? (probe for ease and difficulty)
    f. What are your thoughts on the **format of the tele-coaching CBE intervention** for people living with HIV? (i.e. bi-weekly online coaching sessions, working out at home, receiving coaching at home, exercising x3/week for an hour etc.)

6) How confident are you that other community-based organizations (other YMCAs or other community organizations) may be ***willing and able to offer*** this tele-coaching intervention as a fully developed program?

   a. What do you think will be the greatest barriers to other sites or organizations adopting this tele-coaching intervention? Do you have any ideas on how to overcome these barriers?

7) Do you have any suggestions for ways in which to ***sustain the tele-coaching CBE program*** over the long term in the HIV community?

8) How ***useful*** do you think a CBE intervention such as this one is for people living with HIV?

9) Do you have any suggestions for ways in which to ***continue the tele-coaching CBE program*** over the long term in the HIV community?

10) Do you have suggestions on ***other partnerships or stakeholders*** that should be considered in the long term sustainability of a CBE program? If so, who are they?

Tele-coaching Technology

11) ***a) CBE Coaches:*** What was your ***experience coaching participants through tele-coaching***?
    b) Other CBE Stakeholders: What are your **thoughts on the exercise intervention** being implemented through tele-coaching technology?
    a. Participants ability to use the technology
    b. Satisfaction with technology
        i. Quality (visual, sound etc.)
        ii. Effectiveness of delivering coaching sessions
        iii. Coaching engagement through technology
        iv. Ability to understand coaches instructions through the technology
    c. Reliability of the technology
        i. Online technology connection
        ii. Confidence in technology to deliver coaching sessions
        iii. Interruptions
        iv. Responsiveness of technology
        v. Confidentiality or privacy concerns
    d. Feasibility
        i. Concerns with implementation of technology for online coaching sessions and group classes.
    e. Sustainability of tele-coaching and online classes following completion of the study

Summary

Is there anything else you would like to say about translating the online tele-coaching CBE intervention? Thank you very much for participating in this interview today. Your responses will help to provide a better understanding of the translation of CBE programs with the HIV community and will help to formulate a future programs down the road.
